# Fitness parameters as a marker of cardiac autonomic neuropathy among diabetes mellitus patients

**DOI:** 10.6026/973206300221564

**Published:** 2026-03-31

**Authors:** Jagrati Nagar, S.M Hulke, G.A Birajdar, A.E Thakare, R.N Bharshankar, Santosh Wakode, Y.P Vaidya

**Affiliations:** 1 Department of Physiology, All India Institute of Medical Sciences, Bhopal, India; 2 Department of Physiology, Parbhani Medical College (PMC), Parbhani, Maharashtra, India; 3 Department of Anatomy, Peoples Medical College and Research Centre, Bhopal, India

**Keywords:** Cardiac autonomic neuropathy (CAN), diabetes mellitus, fitness assessment

## Abstract

The role of fitness parameters as a marker of cardiac autonomic neuropathy in the diagnosis of diabetes mellitus is of interest. Hence, a cross-sectional study was conducted at AIIMS, Bhopal, on 110 diagnosed cases of diabetes mellitus, where patients had undergone fitness assessment and autonomic function assessment using Ewing's battery of autonomic reactivity and heart rate variability assessment. A significant difference was observed in cardiovascular endurance and abdominal strength between early to regular involvement of cardiac autonomic neuropathy and definite to severe involvement. However, no significant correlations were observed between fitness parameters, the autonomic function involvement score and heart rate variability parameters. Cardiovascular endurance may act as an early marker of cardiac autonomic neuropathy in diagnosed cases of diabetes mellitus; thus, fitness assessment may be recommended for diabetes mellitus patients. However, longitudinal studies are recommended in this regard.

## Background:

The International Diabetes Federation projected that the diabetes population in the Southeast Asia region may increase to 185 million by 2050. Prevalence would also reach 13.2 % in the Southeast Asia region. Presently, about 89 million of the Indian population is suffering from diabetes mellitus and the age-standardized prevalence in the year 2024 is about 10.5% [1]. Diabetes mellitus is associated with various complications. Cardiac autonomic neuropathy (CAN) is one of the complications observed in patients with diabetes mellitus. Prevalence of CAN ranged from 17 to 90 percent in type 1 diabetes mellitus patients and 27 to 93 percent in type 2 diabetes mellitus patients. Clinically, cardiac autonomic neuropathy (CAN) may manifest as resting tachycardia, reduced exercise tolerance and orthostatic hypotension. CAN patients may be associated with arrhythmias, silent ischemia, cerebrovascular disease, cardiomyopathy and chronic kidney disease [2]. Cardiac autonomic neuropathy (CAN) may be developed by several mechanisms. Some mechanisms through which it plays a role are hyperglycemia and the renin-angiotensin mechanism [3]. Hyperglycemia induces oxidative stress and produces toxic glycosylation end products, which are considered to be responsible for neuronal dysfunction. Various components of the renin-angiotensin system, such as Angiotensin II, Angiotensin (1-7) and renin, may modulate the sympathetic and parasympathetic nervous systems [4]. Exercise in any form has a protective effect on CAN [5]. It normalizes glucose, increases antioxidants, enhances cardiac vagal activity and decreases angiotensin II activity [6]. All the exercises ultimately have a positive effect on fitness parameters. Fitness parameters make us aware about flexibility, muscular strength, core strength, muscular endurance and cardiovascular endurance. If diabetic patients have good fitness, glycaemic control may be reasonable; thus, it may contribute to cardiac autonomic neuropathy in diabetes mellitus patients [7]. With this background in mind, a study was designed to determine whether fitness parameters can predict cardiac autonomic neuropathy in diabetes mellitus patients. Some studies have been reported in the literature. However, not all fitness parameters are studied [8, 9]. Therefore, it is of interest to assess the role of fitness parameters in the development of cardiac autonomic neuropathy in patients with diagnosed diabetes mellitus.

## Subjects and Methods:

It was a cross-sectional study conducted at AIIMS, Bhopal, which included 110 diagnosed cases of diabetes mellitus in the 30-50-year age group. Department where it was conducted is the Physiology, Medicine and Endocrinology Department, AIIMS, Bhopal. Departmental RRB approval and ethical clearance were obtained prior to the study. Exclusion criteria for the patients were end stage renal disease, a known case of heart disease, uncontrolled hypertension, uncontrolled diabetes mellitus and any neuromuscular disorder not allowing fitness assessment. Autonomic function and fitness evaluation were done after obtaining written informed. All these parameters were measured using standard guidelines [10-11]. Ewing's battery of tests was used for autonomic function evaluation. Lying to standing heart rate response and BP response, Blood pressure response to hand grip test, Valsalva ratio, Heart rate variation with respiration and the cold pressor test were also used. These parameters were assessed using a 16-channel Polygraph system (Gentech), a Motorized tilt table (Medica Podium) and B.P. measurement using the Diamond model (BPDG 124). Standard methods were used to conduct these test [12]. Fitness assessment parameters were measured. The parameters and methods measured are listed in Table 1 (see PDF).

## Heart rate variability:

HRV recording and assessment were done using a 16-channel Polygraph system (Gentech). Sampling was done at 1000 HZ for 6 minutes. The recording was done in a quiet room with a comfortable temperature. It was ensured that the patient was stable prior to recording. Heart rate variability analysis was done using Kubios software. Interpretation was done based on Ewing's battery of test. Standardized guidelines were followed for interpretation and procedure [12, 13- 14]. A statistical analysis was conducted using simple linear regression analysis and Spearman correlation analysis to investigate the correlation between fitness parameters, autonomic function involvement score and heart rate variability parameters. An unpaired t-test was used to compare the fitness parameters in early to regular involvement of cardiac autonomic neuropathy vs. definite to severe involvement of cardiac autonomic neuropathy

## Results and Discussion:

The study was conducted in 70 male (Age, mean± S.D. 40.9 years ± 7.76, Height, mean± S.D. 166.36 cms ± 6.48, weight, mean± S.D. 72. 8 Kg ± 10.61) and 40 female (Age, mean± S.D. 43.78 years ± 7.08, Height, mean± S.D. 150.99 cms ± 6.38, weight, mean±S.D. 64.17 Kg ± 12.98). Duration of diabetes was (mean± S.D., 3.34 years ± 1.24). Forty-nine patients had normal to early involvement of autonomic function, while 61 patients showed definite to severe involvement (Figure 1 - see PDF). Heart rate variability parameters of the diabetes patients are displayed in (Table 2 - see PDF). Fitness parameters were compared in these two groups as shown in (Table 3 - see PDF). A significant difference was observed for cardiovascular endurance and abdominal strength, early to regular involvement of cardiac autonomic neuropathy versus definite to severe involvement of cardiac autonomic neuropathy. Correlation between fitness parameters and autonomic function testing scoring is displayed in (Table 4 - see DF). CAN parameters and fitness parameters does demonstrate significant correlation. Through this study, we have assessed the role of fitness parameters in developing cardiac autonomic neuropathy in newly diagnosed cases of diabetes mellitus. We had compared various fitness parameters, from normal to early versus definite to severe involvement of cardiac autonomic neuropathy. We only had significant results for cardiovascular endurance and abdominal strength. There was no significant correlation between fitness parameters and CAN parameters. This study is unique in that all fitness parameters were assessed. All these fitness parameters help us know the individual's flexibility, muscular strength, core strength, muscular endurance and cardiovascular endurance status. There are some studies where CAN has been linked to a reduction in cardiorespiratory fitness [15]. This is similar to our findings. Hence, various aerobic exercise programs are prescribed in diabetes mellitus patients, improving aerobic power and having an overall positive effect on cardiac autonomic neuropathy, as shown by numerous studies [16, 17]. Cardiorespiratory fitness was considered to be a stronger predictor of mortality than established risk factors such as smoking, hypertension, high cholesterol and type 2 diabetes mellitus and the addition of cardiorespiratory fitness to traditional risk factors may alter the risk for adverse outcomes [18]. Association between muscle performance and cardiac autonomic neuropathy were evaluated by some. Cardiac autonomic neuropathy was assessed by heart rate variability. The author found decreased muscle performance, hamstring flexibility and heart rate variability in patients with diabetes mellitus [19]. But they also found a nonsignificant correlation, as observed by us. Decreased abdominal strength was observed in patients with definite to severe involvement of cardiac autonomic neuropathy in the present study; however, this difference was not significant for flexibility. Various studies detailing fitness assessments in patients with diabetes mellitus, which show that their fitness is lower compared to their age- matched controls [20]. The presence of diabetes mellitus and hypertension was associated with lower grip strength among individuals with a healthy BMI [21]. However, there are limited studies that examine fitness parameters in different grades of cardiac autonomic neuropathy. Cardiac autonomic neuropathy may affect fitness in diabetes mellitus patients. Cardiovascular endurance adaptation occurs with aerobic exercise training. This adaptation depends on the maximum stroke volume attained, cardiac output distribution and blood flow to active muscles. Blood flow to active muscle would directly affect nutrient transport and delivery to exercising muscle [22]. The autonomic nervous system plays a vital role in these adaptations. Diabetes mellitus is associated with various microvascular complications and cardiac autonomic neuropathy may disrupt all these factors. CAN is associated with decreased vagal tone and impaired baroreflex sensitivity [23]. All these factors may contribute to decreased cardiovascular endurance, as observed in the present study. Additionally, other factors that contribute to CAN in diabetes mellitus may also impact fitness level. One of the primary culprits is hyperglycemia. It increases the production of free reactive oxygen species, which causes oxidative damage. Various mechanisms may be activated, including protein kinase C activation, advanced glycosylation end products and hexosamine activation, which can lead to vascular occlusion, endothelial dysfunction, leakage and inflammation [24]. When a person is fit, their physical activity status is good. Physical activity normalises glucose, increases antioxidants and decreases cytokines like IL-18 [25]. Hence, in our study, cardiovascular endurance was better in individuals with normal to early stages of CAN than in those with definite to severe CAN. Other mechanisms reported in the literature for CAN in the renin-angiotensin mechanism act through various mechanisms to alter the sympathetic, parasympathetic tone and baroreflex sensitivity. A physically fit person directly affects the renin-angiotensin mechanism by action on angiotensin II [26]. However, we could not get a significant change in major fitness parameters in the present study. The reason for this may be the duration of diabetes mellitus, which was around than 3 years and persons with end-stage disease and uncontrolled diabetes mellitus were excluded from the study. The duration required for other fitness parameters might be more than that needed for cardiovascular endurance. Limitations of the studies: The study was cross-sectional; longitudinal studies with an exercise program would give more insight into the relationship between fitness parameters and CAN. Further, studies should be planned with a focus on the molecular mechanisms of exercise, which may play a role in the development/prevention of CAN in diabetes mellitus patients.

## Conclusion:

Fitness parameters, such as cardiovascular endurance and abdominal strength, may serve as an early marker of cardiac autonomic neuropathy in patients with diabetes mellitus; however, longitudinal studies are recommended in this regard.
